# CEHD: A Unified Framework for Detection and Height Estimation of Fresh Corn Ears in Field Conditions

**DOI:** 10.3390/plants15010038

**Published:** 2025-12-22

**Authors:** Hengyi Wang, Yang Li, Jun Fu, Qiankun Fu, Yongliang Qiao

**Affiliations:** 1 College of Biological and Agricultural Engineering, Jilin University, Changchun 130022, China; hengyi23@mails.jlu.edu.cn (H.W.); liyang20210706@163.com (Y.L.); fu_jun@jlu.edu.cn (J.F.); 2 Key Laboratory of Efficient Sowing and Harvesting Equipment, Ministry of Agriculture and Rural Affairs, Jilin University, Changchun 130022, China; 3 Key Laboratory of Bionic Engineering, Ministry of Education, Jilin University, Changchun 130022, China; 4 Australian Institute Machine Learning (AIML), University of Adelaide, Adelaide, SA 5005, Australia; yongliang.qiao@ieee.org

**Keywords:** fresh corn, YOLO-HAMDF, DeepSORT, RGB-D object detection, height measurement

## Abstract

Real-time detection of fresh corn ear height can provide a basis for dynamic adjustment of harvester header parameters, reducing mechanical damage and improving harvest quality. This study proposes a corn ear height detection model (CEHD). A YOLO-HAMDF network is developed for ear recognition, in which the core modules—TBDA, GLSA, and AQE—respectively suppress background interference, enhance contextual perception, and optimize bounding-box scoring. Depth information is incorporated to filter non-target regions and improve system robustness. In addition, a DI-DeepSORT module is designed for ear tracking, where DBC-Net and IDA-Kalman, respectively, enhance the discriminability of ReID features and enable independent-dimension adaptive noise modeling with smoothed positional updates. Experimental results demonstrate that the proposed CEHD model achieves a mean absolute error (MAE) of only 3.21 ± 0.05 cm under field conditions, indicating strong stability and practical applicability. In summary, this study presents a stable and reliable corn ear height detection system, achieves real-time monitoring of ear height, and provides data support for the dynamic adjustment of header parameters in fresh corn harvesters.

## 1. Introduction

Fresh corn is a high-value crop used for both vegetable and grain purposes and holds significant economic importance [1]. However, its kernels typically contain more than 60% moisture at harvest [2]. Their soft texture and low resistance to mechanical impact make them highly susceptible to breakage during mechanized harvesting [3,4], which severely reduces product quality and economic return. With the advancement of agricultural intelligence and digitalization—particularly within the Agriculture 5.0 framework—the real-time and accurate acquisition of crop phenotypic traits, such as ear characteristics, has become an effective means to guide machinery parameter adjustment and reduce harvest-induced damage [5]. Among these traits, ear height is a key phenotypic indicator. Its accurate measurement directly supports the dynamic adjustment of harvester header parameters, thereby minimizing ear damage under complex field conditions [6].

As the primary prerequisite for height measurement, object detection has attracted extensive research interest worldwide. Compared with traditional detection methods such as color-difference analysis [7], K-means clustering [8], fuzzy C-means [9], KNN-based approaches [10], and SVM-based classification [11], deep learning–based object detection techniques offer stronger feature extraction capabilities and markedly improved real-time performance. Current detection frameworks are primarily based on Transformer architectures and convolutional neural networks (CNNs). Representative Transformer-based approaches include Vision Transformer [12], Swin Transformer [13], and RT-DETR [14]. Wu et al. developed CBAM-RT-DETR, a real-time detector that integrates CBAM and grouped convolutions to enhance shallow features, improving maize seedling detection in complex field environments [15]. Simşek Bagcı et al. proposed a crop-recognition method based on a Deep Transformer Encoder that uses multi-temporal Sentinel-1 and Landsat-8 imagery to automatically identify cotton and corn planting areas, achieving 85–95% classification accuracy across different temporal combinations [16]. Zhang et al. introduced an MConv-SwinT–based maize quality detection model that fuses shallow and deep features and incorporates a convolutional block attention mechanism. The model achieved 99.89% accuracy in classifying maize quality levels [17]. Although Transformer-based models effectively integrate multi-scale information and handle occlusions, their strong emphasis on long-range dependencies often results in the loss of shallow detail information. In addition, their high computational cost and large parameter size pose major challenges for embedded deployment and real-time applications [18]. In contrast, lightweight CNN-based detectors provide efficient and stable performance in resource-constrained environments while maintaining high computational efficiency [19]. Among CNN-based detectors, the YOLO family has become highly influential due to its strong real-time performance, high accuracy, and compact architecture. For instance, Gai et al. integrated DenseNet into YOLOv4 and designed circular annotation boxes to detect cherries at different maturity stages with high precision [20]. Chen et al. proposed GE-YOLO based on YOLOv8, leveraging a Gold-YOLO structure and EMA attention to enhance the robustness of rice-weed recognition [21]. Zhou et al. introduced YOLO-ACE, which incorporates a contextual enhancement module and selective convolutional attention to improve cotton-field weed detection [22]. Sun et al. proposed ESC-YOLO by integrating EfficientViT with SCConv, improving apple fruit recognition performance [23]. Considering the requirements for real-time performance and embedded deployment, this study adopts the lightweight and mature YOLO11-L as the baseline model for corn ear detection.

However, two-dimensional object detection provides only planar information and cannot obtain the height measurements required for adjusting harvester header parameters. Depth cameras, as common sensors for acquiring three-dimensional visual information, are insensitive to lighting conditions, robust to complex backgrounds, and capable of providing highly stable depth data [24]. When integrated with object detection, they allow reliable estimation of the three-dimensional position of corn ears, thereby enabling accurate ear-height measurement. Zhong et al. combined Faster R-CNN with depth information to predict pepper plant height, achieving an error within 4.4 mm [25]. Baden Parr et al. utilized RGB and ToF depth cameras together with YOLO7 to detect grape berries and estimate their three-dimensional characteristics, obtaining a counting correlation coefficient of 0.946 [26,27]. Zhao et al. integrated an improved YOLO8n-seg model with depth information to measure lettuce height, achieving accuracies of 94.339% in hydroponic systems and 91.22% in potted scenarios [28]. These studies demonstrate notable progress in crop-height measurement.

Most height-measurement studies still focus on improving object detection, with insufficient attention given to object tracking. The stability of the detected bounding boxes directly affects the accuracy of depth information extraction [29]. Introducing tracking algorithms can therefore further improve the accuracy and stability of height estimation. Existing studies have shown that integrating object detection with tracking can yield strong performance: Wang et al. integrated YOLO with SORT for mango tracking and counting [30]; Li et al. combined YOLO8 with ByteTrack to build a real-time maize kernel-shedding counting system [31]; Ye et al. integrated an improved YOLO5 with StrongSORT, employing attention mechanisms and weighted box fusion to achieve high-precision tracking and counting of densely infected trees [32]; Tu et al. applied lightweight YOLO5s with an improved DeepSORT, utilizing trajectory delay and secondary IoU matching to enable robust fruit detection, tracking, and counting in complex environments [33]. However, different tracking algorithms exhibit notable limitations. SORT relies solely on motion cues, making it prone to ID switching in occlusion or dense-target scenarios. StrongSORT increases computational complexity and is less suitable for embedded deployment. ByteTrack improves recall for missing detections but struggles to distinguish small, visually similar targets [34]. In contrast, DeepSORT integrates motion and appearance cues, providing a favorable balance between ID consistency and computational efficiency, and thus serves as a strong baseline for further improvement. Currently, tracking-algorithm enhancements specifically targeting crop height measurement remain limited. Most existing studies employ general-purpose tracking models, which do not fully complement the enhanced detection modules and thus fail to form a specialized height-measurement system tailored to the characteristics of specific crops and field environments.

Complex backgrounds and color-similar interference can significantly reduce object detection accuracy in fresh corn harvesting scenarios. Occlusion and the close spatial arrangement of ears often blur ear boundaries and cause false detections. Ears located farther from the camera appear smaller and are more susceptible to missed detections and spurious high-confidence boxes. Harvester-induced motion introduces positional fluctuations across consecutive frames, leading to discontinuous detection outputs and reduced height-measurement accuracy. To address these challenges, a dedicated corn ear dataset was constructed. Unlike public datasets, it simulates real harvesting conditions in both camera angle and installation height, and simultaneously records depth information aligned with RGB images. Based on this dataset, a corn ear height detection framework integrating YOLO-HAMDF and DI-DeepSORT was developed. High-precision ear localization under complex field conditions is achieved through three modules: the TBDA module, which incorporates multi-scale dynamic convolution and triple-attention fusion; the GLSA module, which enables collaborative modeling of local and global features; and the LQE, which integrates occlusion cues and dynamic score suppression. In addition, the DBC-Net architecture and the proposed IDA-Kalman algorithm enhance the DeepSORT framework, improving tracking stability and accuracy. Combined with depth information, the overall framework provides robust ear detection and reliable height estimation in challenging harvesting environments. The main contributions of this study are as follows: (1) A YOLO-HAMDF model was designed, significantly improving corn ear recognition accuracy under complex field conditions; (2) A DI-DeepSORT model was introduced to address the limited feature-extraction capacity and non-adaptive observation-noise modeling of traditional DeepSORT, thereby improving cross-frame tracking consistency; (3) A corn ear height detection model (CEHD) was developed by integrating depth information, enabling real-time monitoring of ear height in the field.

## 2. Experimental Results and Analysis

### 2.1. Ablation Study

To evaluate the contribution of each module to corn ear height detection, ablation experiments were conducted on the self-constructed fresh corn ear dataset (FCE-GBD Dataset). YOLO11-L combined with the traditional DeepSORT was used as the baseline, and each module as well as their combinations were tested multiple times. Five random seeds were applied for each experiment to assess result stability, with both mean values and standard deviations reported. The results are summarized in Table 1.

For detection accuracy (mAP0.5), introducing individual modules TBDA, GLSA, and AQE increased mAP0.5 to 96.35 ± 0.12%, 96.19 ± 0.15%, and 95.21 ± 0.10%, respectively, with TBDA yielding the largest improvement. For pairwise combinations, mAP0.5 reached 96.80 ± 0.10% for TBDA + GLSA, 96.75 ± 0.10% for TBDA + AQE, and 96.90 ± 0.09% for GLSA + AQE, demonstrating the synergistic effect of modules. The three-module combination (TBDA + GLSA + AQE) achieved the highest mAP0.5 of 97.01 ± 0.08%, which remained unchanged when all modules were employed simultaneously.

In terms of detection error (MAE) and correlation (Pearson r), single-module MAEs were 5.26 ± 0.08 cm (TBDA), 5.19 ± 0.10 cm (GLSA), 4.15 ± 0.07 cm (AQE), and 5.56 ± 0.09 cm (DI-DeepSORT). Pairwise combinations yielded MAEs of 4.90 ± 0.07 cm (TBDA + GLSA), 4.05 ± 0.06 cm (TBDA + AQE), and 4.00 ± 0.05 cm (GLSA + AQE), with Pearson r ranging from 0.87 to 0.91 ± 0.01. The three-module combination achieved an MAE of 3.50 ± 0.05 cm and Pearson r of 0.92 ± 0.01, while the full-module combination yielded the lowest MAE of 3.21 ± 0.05 cm and the highest Pearson r of 0.94 ± 0.01.

Regarding inference efficiency, introducing single modules had minimal impact on FLOPs (87.1–87.4 G), inference latency (32.8–33.5 ms), and frame rate (30–31 FPS). When all modules were integrated, FLOPs increased to 88.3 G, latency rose to 36.5 ms, and frame rate decreased slightly to 28 FPS. These results demonstrate a balance between high accuracy and real-time performance suitable for field deployment.

Specifically, the three improved modules in YOLO-HAMDF each serve distinct functions. The TBDA module employs multi-scale dynamic convolution combined with a triple attention mechanism—SE channel attention, spatial attention, and energy function attention—to fuse features across different receptive fields. This design enables the model to capture both fine-grained variations and spatial structural characteristics of corn ears while emphasizing foreground information and suppressing background interference from leaves and weeds. As a result, adaptability and bounding-box stability are significantly enhanced under complex scenarios. The GLSA module splits input features into local and global branches. The local branch leverages depthwise separable convolutions, residual connections, and attention mechanisms to strengthen detailed representation in key regions of the corn ears. The global branch captures long-range spatial dependencies and supplements global semantic information. Together, these branches optimize feature representation and contextual awareness. The AQE introduces an occlusion factor, dynamic output, and a staged score fusion strategy. It effectively suppresses falsely high-confidence boxes for occluded or distant targets, reducing misdetections and false positives. The staged fusion strategy applies additive fusion during early training and transitions to multiplicative fusion in later stages, improving training convergence and enhancing suppression of low-quality targets. Collectively, the three modules enable the model to achieve higher robustness and precision in dense, occluded, and scale-diverse field environments. They provide a stable and reliable foundation for corn ear localization and height measurement.

Furthermore, the DI-DeepSORT plays a critical role in corn ear height detection. For feature extraction, DBC-Net replaces the original CNN and employs multi-scale convolution combined with channel–spatial attention to effectively highlight salient corn ear features, enhancing cross-frame matching robustness. In addition, the traditional Kalman filter is enhanced by breaking the constraint of fixed observation noise modeling. IDA-Kalman dynamically adjusts the observation noise covariance matrix based on detection confidence and per-dimension weighting, improving adaptability to occlusions and low-confidence observations. Moreover, the introduced exponential smoothing mechanism reduces jitter in the center position, further enhancing tracking stability and continuity.

### 2.2. Comparison Experiments

#### 2.2.1. Comparison of Detection Performances

To validate the effectiveness of the proposed method for fresh corn ear detection, a comparative evaluation was conducted on the FCE-GBD Dataset. The improved model was evaluated against 17 mainstream object detection algorithms. These included CNN-based detectors such as Faster R-CNN [35], Cascade R-CNN [36], YOLO5-L [37], YOLO8-L, YOLO11-L [38], and YOLO13-L [39], as well as Transformer-based detectors, including RT-DETRv3 [14] and Swin-T [13]. The results of the comparison are summarized in Table 2.

The experimental results indicate that among CNN-based detection methods, Faster R-CNN and Cascade R-CNN achieved mAP0.5 values of 79.24% and 79.50%, respectively. These models demonstrated relatively low accuracy and high computational complexity (216.3–244.1 G FLOPs). The YOLO series performed better. YOLO5-L, YOLO8-L, YOLO11-L, and YOLO13-L achieved mAP0.5 values of 89.47%, 92.35%, 93.21%, and 94.10%, respectively, while maintaining a modest parameter count of 25.3–46.1 M and FLOPs ranging from 86.9 to 164.8 G. This demonstrates a balance between accuracy and inference efficiency. Transformer-based methods, RT-DETRv3 and Swin-T, achieved slightly higher mAP0.5 values of 91.50% and 92.70% compared with YOLO8-L. However, their larger parameter sizes (52–60 M) and higher computational costs (180–210 G FLOPs) resulted in relatively lower efficiency. The proposed YOLO-HAMDF model introduces TBDA and GLSA modules, as well as the AQE, achieving significant performance improvements. It attained mAP0.5 of 97.01%, mAP [0.5:0.95] of 93.50%, and mAP0.75 of 91.20%, with APS, APM, and APL values of 82.1%, 93.0%, and 97.5% for small, medium, and large targets, respectively. The model contains only 26.4 M parameters and 89.1 G FLOPs, outperforming other comparative models in both accuracy and efficiency, fully demonstrating the advantage of multi-module collaborative enhancement.

**Figure 1:**
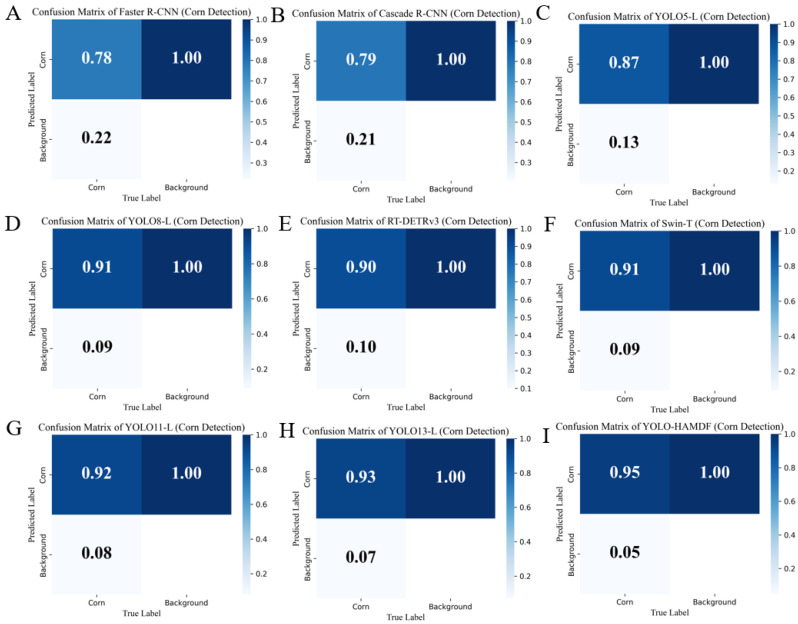
Comparison of confusion matrices of different models evaluated on FCE-GBD. (**A**) Faster R-CNN. (**B**) Cascade R-CNN. (**C**) YOLO5-L. (**D**) YOLO8-L. (**E**) RT-DETRv3. (**F**) Swin-T. (**G**) YOLO11-L. (**H**) YOLO13-L. (**I**) YOLO-HAMDF.

Figure 1 presents a comparison of confusion matrices across all detection models. Among CNN-based models, Faster R-CNN and Cascade R-CNN achieved recall rates of 0.78 and 0.79, indicating limited capability in detecting small, occluded, or sparsely distributed corn ears in complex field environments. YOLO series models performed better. YOLO5-L, YOLO8-L, YOLO11-L, and YOLO13-L achieved recall rates of 0.87, 0.91, 0.92, and 0.93, demonstrating stronger detection ability for multi-scale targets in complex backgrounds. Transformer-based RT-DETRv3 and Swin-T achieved recall rates of 0.90 and 0.91, showing good performance for large targets but slightly weaker recognition for small or sparse corn ears. The proposed YOLO-HAMDF achieved the highest recall rate of 0.95, representing an improvements of approximately 4% and 2% compared with Swin-T and YOLO13-L, respectively. The combination of high recall and low miss rate highlights the robustness and reliability of YOLO-HAMDF for corn ear detection under complex field conditions.

#### 2.2.2. Comparison of Tracking Performances

**Figure 2:**
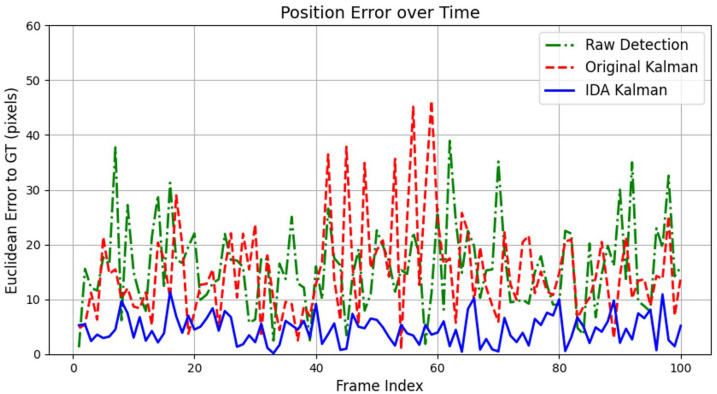
Euclidean position error over frames for corn ear tracking with different filtering strategies.

To objectively evaluate the performance of the improved IDA Kalman filter in corn ear tracking, the tracking results of three methods were compared under identical detection conditions: Raw Detection (direct outputs of the YOLO11 detector), Original Kalman (the conventional Kalman filter), and IDA Kalman (the improved adaptive filter). All tests employed the YOLO-HAMDF model for corn ear detection. As shown in the Euclidean error curves (Figure 2), the Raw Detection method exhibited the largest positional errors, with obvious jitter and noise. The Original Kalman filter partially suppressed detection noise, reducing errors; however, significant fluctuations were still observed during occlusion or low-confidence observation periods (frames 40–60). In contrast, the IDA Kalman filter substantially reduced overall errors and effectively suppressed fluctuations. Quantitative analysis indicates that during the occlusion period, the root mean square error (RMSE) of IDA Kalman was only 3.5 pixels, compared with 8.2 pixels for the Original Kalman filter and 12.0 pixels for Raw Detection.

Furthermore, the proposed method was compared with DeepSORT, ByteTrack, and StrongSORT. To ensure a fair comparison, all four tracking methods were evaluated using the detection outputs from the YOLO11-L detector. As shown in Table 3, the proposed method achieved a MOTA of 92%, outperforming DeepSORT (88%), ByteTrack (86%), and StrongSORT (89%). This indicates that the introduction of the IDA Kalman filter significantly improved the accuracy of target state prediction and association. Regarding the IDF1 metric, the proposed method achieved 90%, higher than DeepSORT (85%), ByteTrack (82%), and StrongSORT (87%). This demonstrates enhanced identity preservation and effectively reduces target drift. The number of ID switches was only 15, considerably lower than DeepSORT (30), ByteTrack (40), and StrongSORT (20), further confirming stable tracking performance under occlusion and dense-target scenarios. Moreover, the proposed method maintained high tracking accuracy while achieving a real-time processing speed of 30 FPS, indicating a well-balanced trade-off between efficiency and performance.

Overall, by integrating motion information, appearance features, and independent-dimension adaptive noise modeling, the proposed method effectively improved corn ear tracking accuracy in complex field environments. Moreover, it enhanced the stability of height estimation, demonstrating a clear performance advantage over conventional DeepSORT, ByteTrack, and StrongSORT.

#### 2.2.3. Comparison of Height Measurement Performances

To comprehensively assess the performance of the corn ear height measurement system, multiple detector–tracker combinations were evaluated. Three object detectors—YOLO-HAMDF, YOLO13, and Swin-T—were selected, together with two trackers, DBC-IDA-Deep-SORT and StrongSORT. The comparative results of these combinations are presented in Table 4. The selection of YOLO13, Swin-T, and StrongSORT was based on the preceding comparative experiments (Section 2.2.1 and Section 2.2.2). Specifically, YOLO13 achieved the best performance among CNN-based detectors, Swin-T performed best among Transformer-based detectors, and StrongSORT demonstrated the strongest tracking capability.

Under the unified use of the CHSM-PCM height estimation method, the performance of each detector–tracker combination is summarized in Table 4. Overall, the detector’s feature representation ability and the tracker’s appearance discrimination jointly determine both the accuracy and real-time performance of height measurement. Among all combinations, YOLO-HAMDF + DBC-IDA-Deep-SORT achieved the best results (MAE 3.21 ± 0.05 cm, Pearson r 0.92 ± 0.01, latency 35.4 ± 0.5 ms, FPS 28 ± 0.4). This superiority mainly stems from the higher localization accuracy of YOLO-HAMDF in complex field backgrounds and the more stable appearance matching provided by DBC-IDA-Deep-SORT, which together maintain better temporal consistency in the height estimation process. When YOLO-HAMDF is combined with StrongSORT, although the re-identification capability is improved, the significantly higher computational cost leads to increased latency and reduced FPS, resulting in a slight rise in MAE. For combinations using YOLO13, the weaker feature extraction limits detection of occluded or distant corn ears, leading to higher height estimation errors (MAE 3.51–3.60 cm). Although Swin-T possesses a strong global attention mechanism, its performance on small or densely distributed corn ears is inferior. Furthermore, its slower inference speed further degrades overall results, yielding the lowest performance among all combinations (MAE 3.91–3.98 cm, FPS < 20).

In summary, the detector primarily determines height-measurement accuracy, while the tracker mainly affects temporal stability and real-time performance. The combination of YOLO-HAMDF and DBC-IDA-Deep-SORT achieves the optimal balance among accuracy, robustness, and speed, making it the most practical and application-ready solution in this study.

### 2.3. Visualization

To further validate the effectiveness of the proposed enhancement modules and the overall framework under complex field conditions, a systematic visualization analysis was conducted from multiple perspectives, including feature attention, detection performance, feature distribution, and spatial filtering.

**Figure 3:**
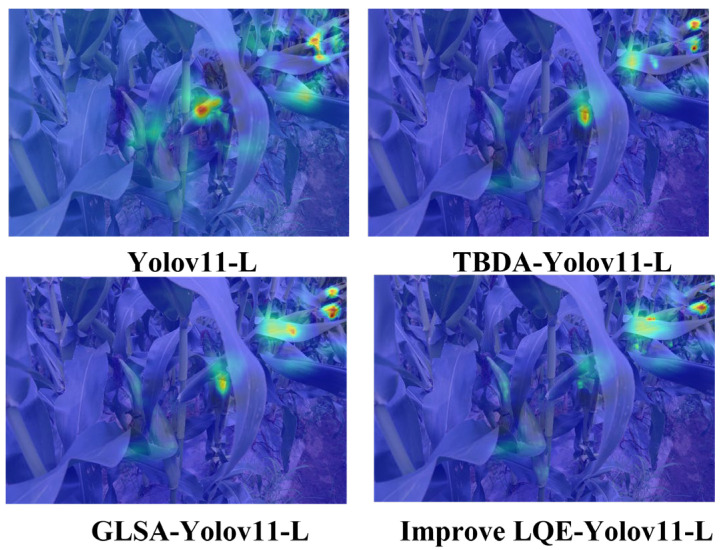
Eigen Grad-CAM visual comparison of feature maps.

First, Eigen Grad-CAM was employed to visualize the feature responses of TBDA, GLSA, and AQE within YOLO11-L (Figure 3). The results indicate that, compared with the baseline YOLO11-L model, the TBDA module substantially reduces attention to leaf regions while markedly enhancing attention to mid- and long-range corn ears. This demonstrates that TBDA effectively suppresses background interference and strengthens target-focused representation. Although the GLSA and the AQE module exhibit slightly weaker suppression of leaf-related responses than TBDA, their heatmaps still show clear improvements over the baseline. Moreover, all three modules display concentrated and continuous attention distributions over target regions, indicating significantly enhanced feature representation capability. These findings collectively support the improved robustness of the model in accurately detecting and localizing corn ears within complex agricultural environments.

**Figure 4:**
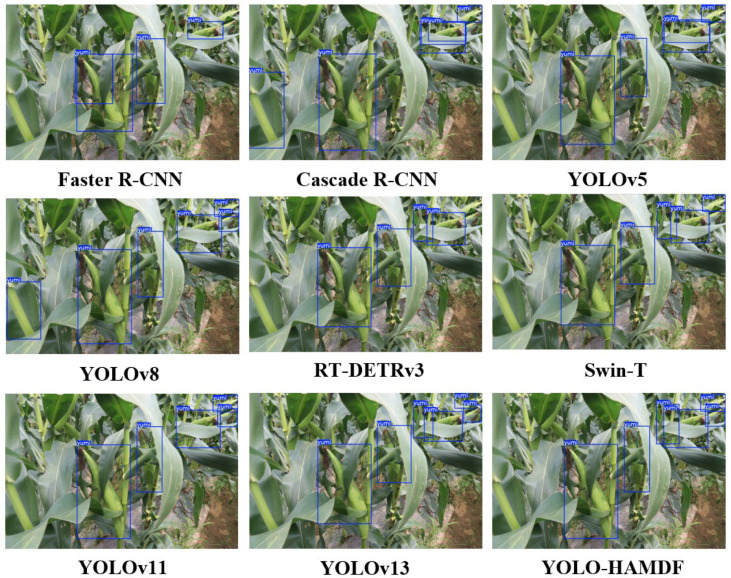
Detection Results of Different Methods.

Based on the module-enhanced model, the performance of various mainstream detection methods was compared on field images of corn ears (Figure 4). Faster R-CNN and Cascade R-CNN exhibited false positives, missed detections, and duplicate detections under conditions of multiple occluded ears and complex backgrounds. YOLO5-L reduced false and missed detections but still produced duplicate detections when ears were occluded. YOLO8-L further mitigated duplicate detections, though false positives and missed detections remained. Transformer-based detectors, including RT-DETRv3 and Swin-T, effectively handled large ears and suppressed background noise, accurately localizing them. However, both models showed missed detections for small or densely occluded ears. Swin-T slightly outperformed RT-DETRv3 in small-ear localization and recognition but remained inferior to the later YOLO models. YOLO11-L achieved notable overall improvements, largely eliminating false and duplicate detections, though missed detections persisted in scenarios with overlapping or small ears. YOLO13-L further enhanced feature extraction over YOLO11-L, nearly eliminating missed detections, with only minor deviations in ear boundary localization. In contrast, the proposed YOLO-HAMDF model demonstrated superior performance in recognizing distant small ears and heavily occluded ears, effectively reducing missed detections while improving localization accuracy.

**Figure 5:**
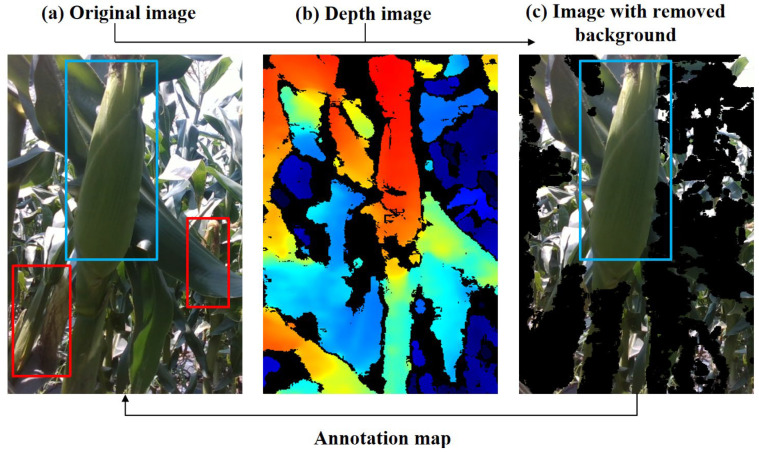
Detection Box Filtering Based on Depth Values. (**a**) Original image. (**b**) Depth image. (**c**) Image with removed background. Red boxes: non-target row ears; blue boxes: target row ears.

The spatial filtering process is visualized in Figure 5. In the original image (Figure 5a), red bounding boxes indicate corn ears in non-target picking rows, while blue boxes denote ears within the target row. Using the depth map acquired by the RGB-D camera (Figure 5b), the spatial distance of each pixel from the camera was extracted. Field measurements show that the row spacing of corn is approximately 0.6 m. Considering that the mobile platform travels along the centerline between rows, a depth threshold of 0.5 m was applied to distinguish target rows from non-target rows. This threshold not only fully includes the plants in the target row but also effectively removes plants and ears in non-target rows beyond 0.5 m. Consequently, irrelevant information is eliminated from the original RGB image, resulting in a background-removed image that retains only the ears in the target row (Figure 5c).

**Figure 6:**
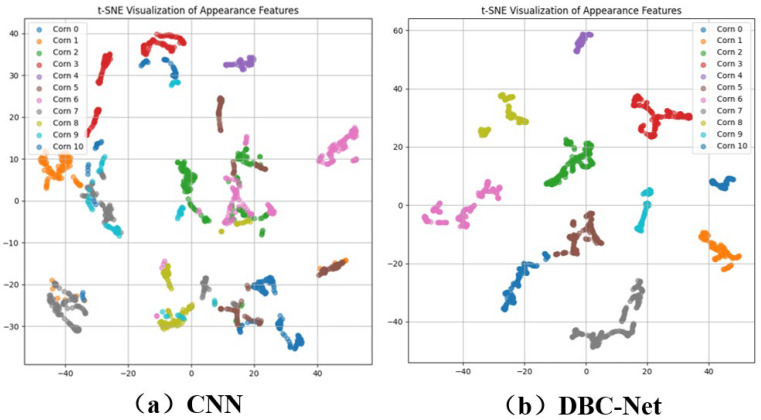
Visualization of the extracted appearance features of corn ears. (**a**) CNN outputs visualization results of corn ear appearance features (**b**) DBC-Net outputs visualization results of corn ear appearance features.

In the target-tracking section, t-SNE visualization was applied to features extracted by the original CNN module and the proposed DBC-Net module (Figure 6). The results show that the original CNN has limited discriminative ability for corn ear appearance features in complex field environments, with feature distributions of different ears largely overlapping. In contrast, DBC-Net significantly improves feature separability, producing more compact clusters for the same ear and greater separation between different ears, thereby providing a robust basis for stable tracking using DI-DeepSORT.

**Figure 7:**
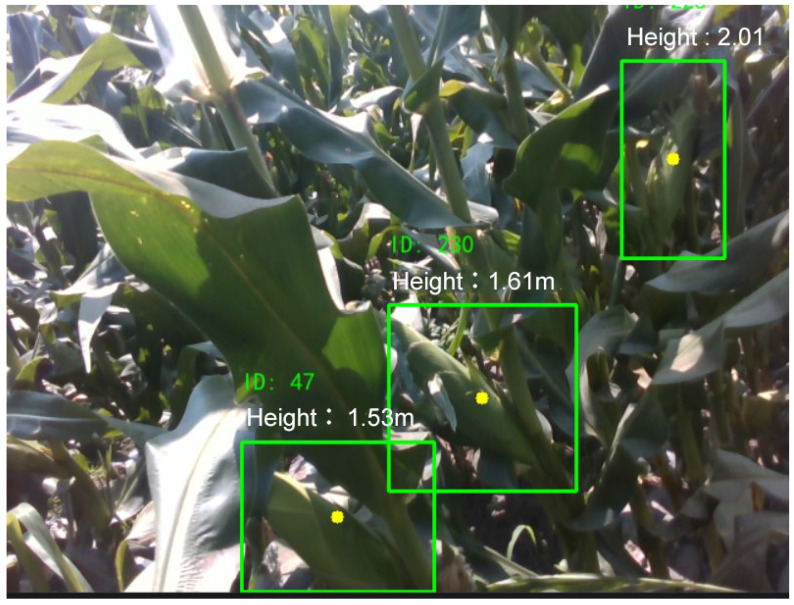
Height Detection Result. Green boxes: detected targets.

The modules function synergistically to form the corn ear height measurement framework. This system not only achieves spatial focus on the target planting rows but also demonstrates higher temporal stability in height estimation. The resulting height measurements are shown in Figure 7, further validating the reliability and practical applicability of the framework in real-world field environments.

## 3. Discussion

This study developed a corn ear height measurement model based on YOLO-HAMDF and DI-DeepSORT, effectively addressing challenges in field environments such as severe occlusion, complex backgrounds, and large variations in ear size. The model enhances ear localization accuracy through the TBDA, GLSA, and AQE modules. Depth information is employed to filter non-target regions, and, combined with DI-DeepSORT, stable height sequences are obtained for each ear, providing reliable input for subsequent mechanized operations.

However, the experiments were primarily conducted in fresh corn field scenarios and did not include systematic benchmark testing on large-scale general-purpose datasets such as COCO. Although YOLO-HAMDF demonstrates excellent performance in agriculture-specific tasks, its generalizability remains to be further validated. Future work will conduct benchmark evaluations on public datasets like COCO according to mainstream computer vision standards. Independent ablation studies of the TBDA, GLSA, and LQE modules across tasks and datasets will also be conducted to comprehensively assess their transferability and structural contributions, thereby strengthening the model’s novelty and generalizability.

In practical applications, the model’s output “height sequences” are not intended for direct point-wise control of harvester parameters. Instead, they are used to construct smoothed and robust dynamic adjustment strategies for the cutter bar. The automatic corn ear detection and height measurement model provides real-time continuous distributions of ear height. By applying sliding-window fusion, trend filtering, and stability evaluation, control signals suitable for the actuator are generated, enabling adaptive adjustment of the cutter bar. This strategy prevents frequent mechanical oscillations while optimizing operational parameters according to real-time field information, demonstrating clear engineering value. Future work will establish a mapping model between corn ear height distributions and cutter bar adjustment parameters, deploying it on edge devices to achieve closed-loop control of harvester operations.

## 4. Materials and Methods

### 4.1. FCE-GBD Dataset

**Figure 8:**
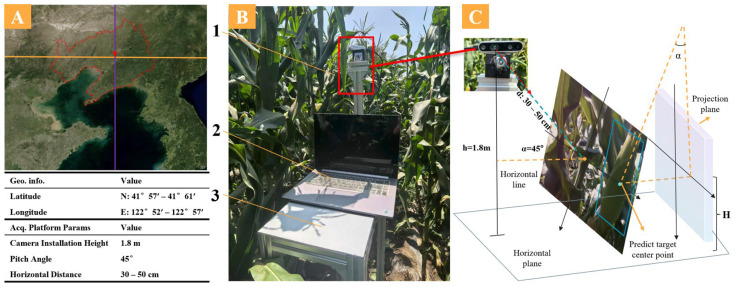
Test area and image acquisition platform. (**A**) Test area. (**B**) Fresh waxy corn image acquisition mobile platform 1. D435i depth camera 2. Computer 3. Mobile car. (**C**) Principle Schematic of Depth Camera.

The study was conducted at a fresh corn planting site in Shenyang, Liaoning Province, China, as shown in Figure 8A. Image data were collected over eight sessions from July to August 2025, covering different dates, eight independent plots, and various lighting conditions—including sunny, cloudy, and backlit afternoons—to ensure sufficient temporal, spatial, and illumination diversity. RGB and depth images were simultaneously captured using an Intel RealSense D435i depth camera (Intel Corporation, Santa Clara, CA, USA), which operates over a range of 0.2–3 m with a depth measurement error of less than 2% within 2 m. The fresh corn image acquisition platform and its schematic are shown in Figure 8B,C. The platform comprises a field-walking device, the D435i depth camera, and a laptop (Lenovo Group Ltd., Beijing, China), with the camera mounted on a gimbal fixed to the walking device. To simulate actual harvester operating conditions, the camera was installed at a height (h) of 1.8 m, with a pitch angle (α) of 45°, and positioned at a horizontal distance (d) of approximately 30–50 cm from the crop row centerline. The variable H represents the measured height of corn ears under these conditions.

**Figure 9:**
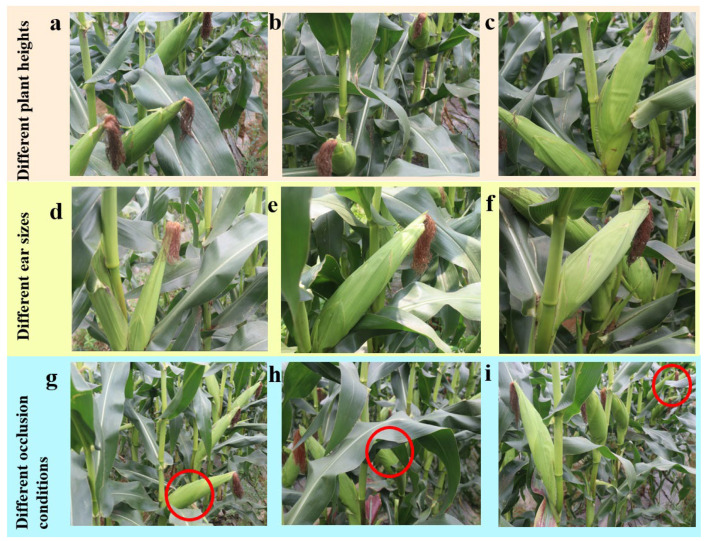
Images of fresh corn under varying growth conditions. (**a**) Low ear, (**b**) Medium-height ear, (**c**) High ear, (**d**) Small ear, (**e**) Medium-size ear, (**f**) Large ear, (**g**) Fully exposed ear, (**h**) Occluded ear, (**i**) Distant and occluded ear.

Significant variations in corn plant height and leaf density were observed across the plots. In some plots, dense foliage partially occluded the ears, whereas sparse leaves in other plots left ears more exposed. These variations increase the difficulty of accurate detection. To enhance the generalization and robustness of the model under diverse field growth conditions, images were collected to cover a range of plant heights, ear sizes, and occlusion scenarios, as illustrated in Figure 2. In Figure 9g, ears are fully exposed, facilitating detection; in Figure 9h, ears are partially occluded by leaves, increasing detection difficulty; and in Figure 9i, distant ears appear smaller with greater overlap and occlusion, posing further challenges. A total of 4000 raw RGB images and corresponding depth maps were collected, encompassing diverse growth conditions.

All images were manually annotated using LabelImg software (version 1.8.4) to obtain ground-truth positions of fresh corn ears. A standardized annotation protocol was followed: only the visible main regions of ears were labeled; heavily occluded ears that remained partially recognizable were annotated according to their visible portions; objects that were entirely invisible or had minimal visible parts, preventing confirmation of target attributes, were excluded. All annotations were cross-checked by a second annotator to ensure consistency and accuracy of bounding boxes.

The dataset was divided into training, validation, and test sets at an 8:1:1 ratio, containing 3200, 400, and 400 images, respectively. Splitting strictly adhered to a sequence-independent principle, ensuring that images from the same plot or captured consecutively on the same day did not appear in different subsets, thereby preventing data leakage. The resulting dataset was named the FCE-GBD Dataset.

### 4.2. CEHD

**Figure 10:**
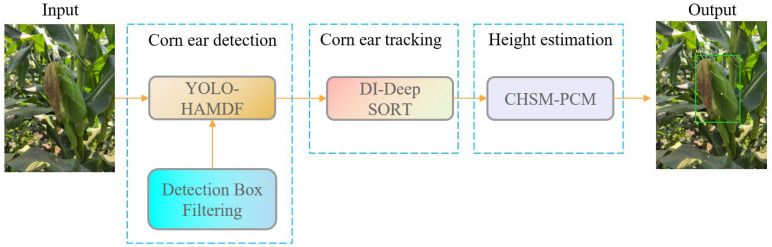
The architecture of CEHD. Green boxes: detected targets.

The framework of the proposed corn ear height detection (CEHD) is illustrated in Figure 10. Images are first input into the YOLO-HAMDF network for ear detection, and non-target ears in adjacent rows are filtered using depth maps to reduce false positives. The filtered bounding boxes are then passed to the DI-DeepSORT module to achieve continuous and stable cross-frame tracking of individual ears. For each tracked target, the CHSM-PCM module computes the local point cloud by back-projecting depth pixels within the predicted bounding box, removing outliers, and calculating the median height. Combined with the camera′s extrinsic parameters, the absolute height of the ear relative to the ground is obtained. Through the coordinated processes of detection, tracking, and height estimation, the framework enables high-precision recognition and reliable height measurement of corn ears under complex field conditions.

### 4.3. YOLO-HAMDF

#### 4.3.1. YOLO-HAMDF Architecture

**Figure 11:**
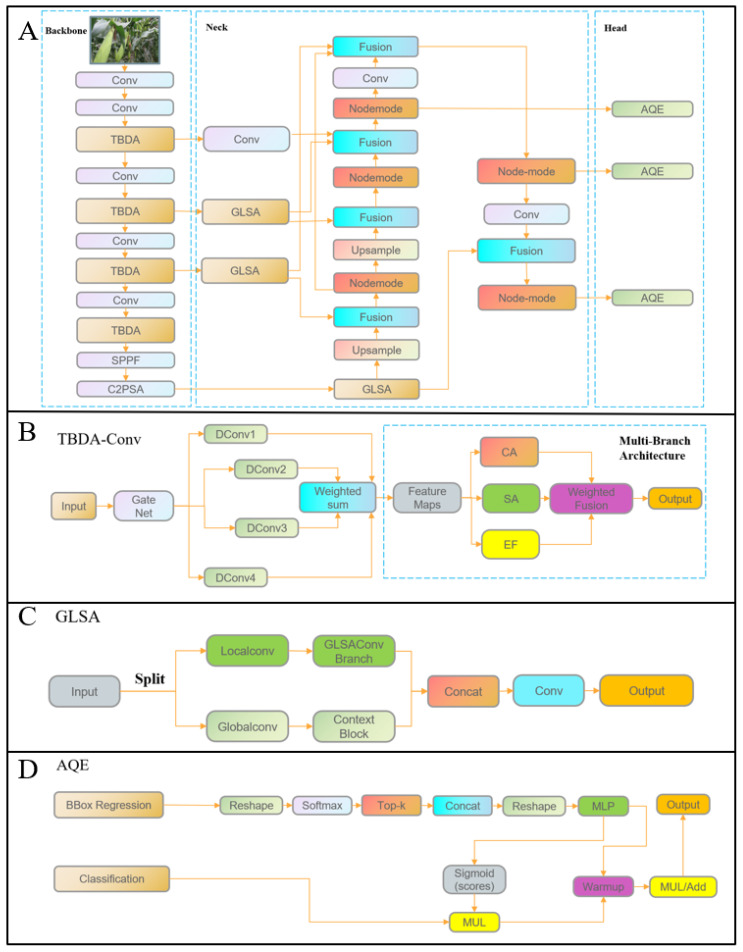
Architecture of the proposed YOLO-HAMDF. (**A**) Overall architecture. (**B**) Architecture of TBDA. (**C**) Architecture of GLSA. (**D**) Architecture of AQE.

The network framework was designed based on YOLO11-L with several efficient module innovations, resulting in the proposed YOLO-HAMDF network. The overall architecture is illustrated in Figure 11. An innovative TBDA module was introduced to replace the original C3 module in the backbone. By combining multi-scale convolutional branches with channel attention, this module effectively enhances the capture of ear details at different scales. To further strengthen feature representation, a GLSA module was incorporated into the Neck, replacing the original YOLO11-L Concat + C3k2 modules with Fusion + Node-Mode + GLSA modules, which were stacked three times at each scale. Finally, the traditional detection heads were replaced with the AQE module to better integrate with depth camera data for corn ear height estimation. The detailed construction of the network is described in the following sections.

#### 4.3.2. TBDA

**Figure 12:**
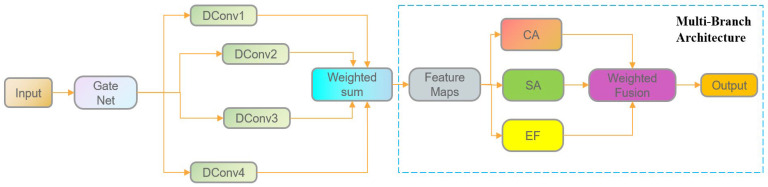
Architecture of TBDA.

Attention mechanisms allow networks to focus on critical features, thereby enhancing object recognition. To address challenges posed by complex background interference in corn ear height detection, the TBDA module was designed. It integrates multiple attention mechanisms to strengthen feature representation and improve detection accuracy, as shown in Figure 12.

The module first introduces a dynamic convolution structure based on a gating mechanism to enable adaptive modeling of multi-scale features. It contains four convolutional branches with kernel sizes of 1 × 1, 3 × 3, 5 × 5, and 7 × 7. A lightweight gating network dynamically generates weight coefficients for each branch. Specifically, global average pooling is applied to the input feature map to extract contextual information, followed by a 1 × 1 convolution and a Sigmoid function to produce branch-specific weight responses. The convolution outputs of all branches are then combined via weighted fusion along the channel dimension, allowing selective enhancement across different receptive fields.

A three-branch attention structure further strengthens feature representation. The first branch uses a channel attention (CA) module to emphasize important semantic channels and suppress irrelevant or noisy channels, enabling the model to focus on spectral and textural features critical for corn ears. The second branch employs a spatial attention (SA) module to highlight regions with strong responses, reinforcing target areas and suppressing background interference, thereby improving localization precision and spatial sensitivity. The third branch applies an energy function (EF) to generate spatial weights based on statistical differences, adaptively amplifying locally salient features and enhancing discriminability under complex backgrounds. Finally, the outputs of the three branches are fused using learnable parameters, providing complementary enhancement across channel, spatial, and energy information and further improving detection performance.

#### 4.3.3. GLSA

**Figure 13:**
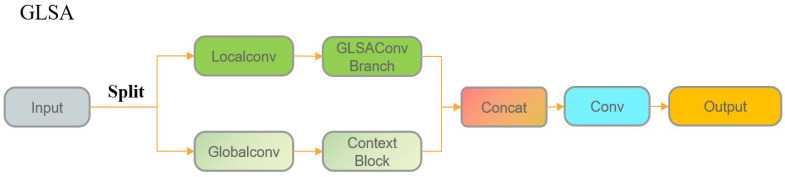
Architecture of GLSA.

From an overall perspective, although the backbone integrates the innovative TBDA convolution with efficient modules such as SPPF and C2PSA to enhance high-level semantic feature representation and suppress background interference, it still struggles to capture long-range dependencies. In complex field scenarios, the network remains susceptible to background distractions, occlusions, and target deformations. To address this issue, the Global-Local Selective Aggregation (GLSA) module was introduced (Figure 13) to enhance feature representation at the Neck stage, thereby improving the localization and recognition accuracy of corn ears. The GLSA module is deployed at the forefront of three detection branches—P3 (low-to-mid level), P4 (mid-to-high level), and P5 (high level)—so that each branch receives enhanced GL features before entering the fusion and decoding paths, effectively improving the perceptual and discriminative capacity of the base features.

Within the GLSA module, the input feature channels are divided into two branches: the Local branch employs a lightweight MLP-style GLSA Conv Branch to model fine-grained features such as local textures and edges; the Global branch leverages a context block based on contextual attention to aggregate global contextual information, complementing the spatial-structural semantics of the target. The outputs of the two branches are then concatenated along the channel dimension and integrated through a convolution to produce a unified output.

The semantic features enhanced by the GLSA module provide stable support for the subsequent LQE, which is based on boundary quality estimation, thereby improving the accuracy and reliability of predicted bounding boxes. By serving as a bridge for global–local information interaction between the backbone and detection heads, the GLSA module demonstrates strong adaptability and robustness in real-world field detection scenarios.

#### 4.3.4. AQE

In recent years, LQE had been widely adopted in the field of object detection. It statistically analyzed the probability distribution of bounding box regression, extracted key distribution features, and then employed a multi-layer perceptron to predict a quality score, which was used to adjust and optimize the bounding box ranking in object detection. This approach effectively improved the accuracy and reliability of the predicted bounding boxes. The specific steps were as follows:

First, the predicted bounding box corner point distribution *P* ∈ *R^B^*^×^^(4^^×^*^reg^*^)^^×^*^H^*^×^*^W^* was normalized using *softmax* to obtain the probability distribution *prob* of each regression category at every location.

(1) $$prob=softmax\left(P\right)$$

From the normalized probability distribution, the top *K* highest probabilities are selected, and their mean was computed to serve as the statistical feature *stat* for that location.

(2) $$stat=\left[top-k\left(prob\right),mean\left(top-k\left(prob\right)\right)\right]$$

The statistical feature was then fed into a multilayer perceptron (*MLP*) to generate a single-channel quality score *Q*, which reflected the confidence of the bounding box prediction at that location.

(3) $$Q=MLP\left(stat\right)$$

Finally, the LQE integrated the quality score with the initial classification confidence to refine the final bounding box score *S*′, thereby providing a more accurate measure of detection quality.

(4) $$S^{\prime }=S+Q$$

**Figure 14:**
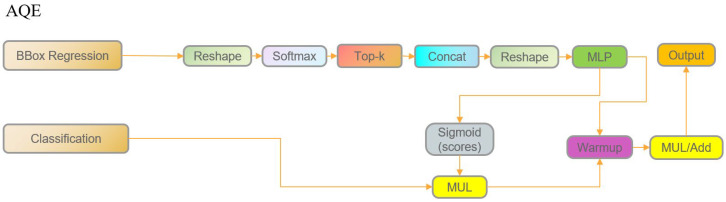
Architecture of AQE.

Through the above process, the LQE not only relied on classification scores but also incorporated statistical information from the regression distribution, thereby effectively improving localization accuracy. However, the original LQE model presented a potential issue when dealing with partially occluded or distant fresh corn ears: such hard-to-classify targets could receive high bounding box quality scores due to relatively accurate localization, even when their classification confidence was insufficient. As a result, these unreliable boxes might still be output by the detector, leading to false detections that not only reduced overall detection accuracy but also hindered subsequent corn height estimation. To address this issue, the LQE module was further enhanced to propose the AQE, with the module architecture shown in Figure 14.

First, an occlusion factor *O* was introduced, which was derived from the initial classification score *S* through a Sigmoid activation:

(5) $$O=\sigma \left(S\right)$$

This factor was employed to adjust the confidence of the bounding box. In cases where the classification score was low but the box quality score was high, the final score of the detection box was reduced to support subsequent height estimation. Specifically, when the detection score did not meet the requirement, the box was temporarily suppressed until occlusion decreased and the classification score increased as the machine advanced. The detection box was finally output only when the overall score *S*′ reached the threshold (empirically *S*′ ≥ 0.75).

In addition, a dynamic adjustment strategy was introduced during training. In the early training stage, an additive approach was adopted to encourage the model to learn as much comprehensive information as possible.

(6) $$S^{\prime }=S+Q\times O$$

In the later stage, after the model had converged, a multiplicative fusion strategy was adopted to more strictly suppress detection responses from low-quality regions.

(7) $$S^{\prime }=S\times \sigma \left(Q\times O\right)$$

This strategy enabled the model to suppress output when detection performance was suboptimal, and to release the corresponding prediction boxes only after the confidence had gradually increased, thereby effectively avoiding overestimation caused by low-quality boxes and enhancing the robustness and reliability of corn ear height detection.

### 4.4. DI-DeepSORT

#### 4.4.1. DI-DeepSORT Architecture

**Figure 15:**
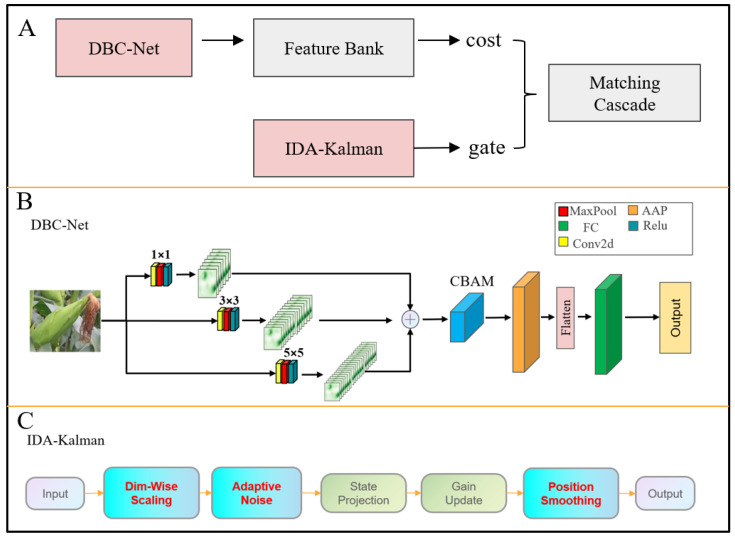
Architecture of the proposed DI-DeepSORT. (**A**) Overall architecture. (**B**) Architecture of DBC-Net. (**C**) Architecture of IDA-Kalman.

In this study, the traditional DeepSORT tracking framework was systematically enhanced, resulting in the proposed DI-DeepSORT (Figure 15). First, to improve the representation of object re-identification (ReID) features, the DBC-Net was designed. This network, based on a dual-line convolution structure combined with a channel–spatial attention mechanism (CBAM), more effectively captures the spatial–spectral features and local details of targets, thereby enhancing the discriminability of multi-object features. Second, to address the sensitivity to noise and position jumps in conventional Kalman filtering for state estimation, the Independent-Dimension Adaptive Kalman Filter (IDA-Kalman) was introduced. This filter adaptively adjusts measurement noise along independent dimensions and applies smoothing to the position center, ensuring stable trajectory estimation under complex conditions. The combined improvements significantly enhance the accuracy and robustness of multi-object tracking with the modified DeepSORT in challenging scenarios.

#### 4.4.2. DBC-Net

**Figure 16:**
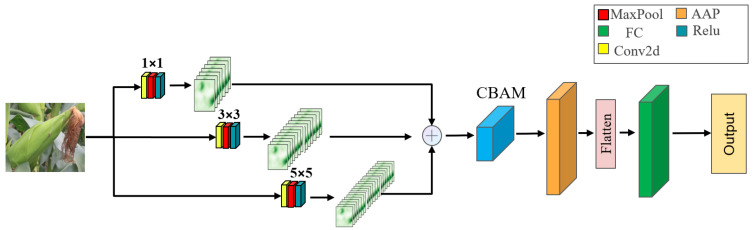
The structure diagram of the DBC-Net in this study.

The DBC-Net architecture is illustrated in Figure 16. In the backbone branch, three convolution operations are sequentially applied with kernel sizes of 1, 3, and 5, and corresponding padding sizes of 0, 1, and 2. Convolutional kernels of different scales extract features from multiple receptive fields: the 1 × 1 convolution primarily compresses and fuses channel information, the 3 × 3 convolution captures fine-grained local features, and the 5 × 5 convolution encodes broader contextual information. This multi-scale convolutional design enables the network to preserve local textural details of corn ears while enhancing its perception of the overall structure. The features obtained from the three convolutional branches are subsequently fused and fed into the CBAM module. Within CBAM, channel and spatial attention mechanisms are used to weight the features. Adaptive average pooling (AAP), a flattening layer, and a fully connected (FC) layer are applied to generate the final feature representation. By integrating local fine-grained details with global structural information, the network achieves a more comprehensive representation of corn ear features.

#### 4.4.3. IDA-Kalman

In the conventional DeepSORT framework, the Kalman filter was used to predict object states and update trajectories, with the target state vector defined as: $x_{k}={\left[x,y,a,h,\dot{x},\dot{y},\dot{a},\dot{h}\right]}^{T}$.

Here, (*x*,*y*) represents the target center position, a denotes the width-to-height ratio, h is the target height, and $\dot{x},\dot{y},\dot{a},\dot{h}$ corresponds to the respective velocities. During the prediction stage, the target state in the current frame was estimated by the Kalman filter based on the state from the previous frame:

(8) $$x_{k\left|k-1\right.}=Fx_{k-1\left|k-1\right.}+B_{uk}$$

(9) $$P_{k\left|k-1\right.}=FP_{k-1\left|k-1\right.}F^{T}+Q$$

*F* is the state transition matrix, *B* is the control matrix, and *Q* is the process noise covariance matrix. When the detector provided new observations, the Kalman filter used these measurements to correct the predicted state:

(10) $$K_{k}=P_{k\left|k-1\right.}H^{T}{\left(HP_{k\left|k-1\right.}H^{T}+R\right)}^{-1}$$

(11) $$x_{k\left|k\right.}=x_{k\left|k-1\right.}+K_{k}(z_{k}-Hx_{k\left|k-1\right.})$$

(12) $$P_{k\left|k\right.=}\left(I-K_{k}H\right)P_{k\left|k-1\right.}$$

*H* is the observation matrix, *R* is the observation noise covariance matrix, and *z_k_* represents the observation provided by the detector. In conventional Kalman filtering, *Q* and *R* are typically set as fixed values. However, in practical object detection scenarios, the quality of detector outputs may vary significantly due to changes in illumination, occlusion, and target scale. A fixed R cannot adequately reflect the uncertainty of observations, causing the filter to over-rely on noisy measurements when detection confidence is low, which may result to bounding box instability.

**Figure 17:**
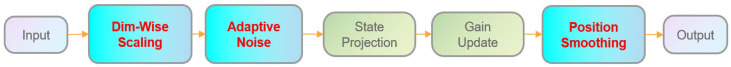
The structure diagram of the IDA-Kalman in this study.

To address the insufficient targeted modeling of measurement noise in conventional Kalman filtering, the Independent-Dimension Adaptive Kalman Filter (IDA-Kalman) was proposed in this study, as illustrated in Figure 17.

This method introduces improvements in measurement noise modeling and position update strategies to enhance the robustness of multi-object tracking in complex scenarios. The observation noise covariance matrix in IDA Kalman was redefined as:

(13) $$R_{t}=diag\left(\sigma_{x}^{2},\sigma_{y}^{2},\sigma_{a}^{2},\sigma_{h}^{2}\right)$$

Here, the measurement noise *σ_i_* for each dimension is dynamically adjusted using the weighted detection confidence $c\in \left[0,1\right]$:

(14) $$\begin{array}{c} \sigma_{x}=max\left(1-c,\varepsilon \right)\cdot w\cdot h_{t-1}\\ \sigma_{y}=max\left(1-0.8c,\varepsilon \right)\cdot w\cdot h_{t-1}\\ \sigma_{a}=max\left(1-0.5c,\varepsilon \right)\cdot 0.1\\ \sigma_{h}=max\left(1-c,\varepsilon \right)\cdot w\cdot h_{t-1} \end{array}$$

*h*_*t*−1_ represents the target height in the previous frame, *w* is the position normalization weight, and ε=0.01 denotes the noise lower bound, preventing the noise from becoming too small as *c* approaches 1. In this study, the measurement noise weights are individually defined for the four state dimensions based on empirical detection performance. Compared with the fixed R in conventional Kalman filtering, IDA Kalman can independently adjust the noise magnitude according to detection confidence and dimension-specific weights, relying more on observations when detection is reliable and more on predictions when detection is uncertain, thereby enhancing the filter’s adaptability to uncertainty. The improved Kalman gain formula is expressed as:

(15) $$K_{t}=P_{t\left|t-1\right.}H^{T}{\left(HP_{t\left|t-1\right.}H^{T}+R_{t}\right)}^{-1}$$

(16) $$x_{t}^{\prime }=x_{t\left|t-1\right.}+K_{t}\left(z_{t}-Hx_{t\left|t-1\right.}\right)$$

*R_t_* has already been dynamically adjusted according to the detection confidence. In addition, to further suppress the jitter of the center position (*x*,*y*), an exponential smoothing mechanism is introduced. After incorporating this mechanism, the expression for *x_t_* is as follows, and *y_t_* is treated similarly:

(17) $$x_{t}=\alpha x_{t-1}+\left(1-\alpha \right)x_{t}^{\prime }$$

$\alpha \in \left[0,1\right]$ represents the smoothing coefficient, with larger values indicating a more stable position.

By employing independent-dimension adaptive noise modeling and smoothing updates of the center position, the filter’s adaptability to observation uncertainty is significantly enhanced, effectively improving the stability and accuracy of multi-object tracking in complex environments.

### 4.5. CHSM-PCM

**Figure 18:**
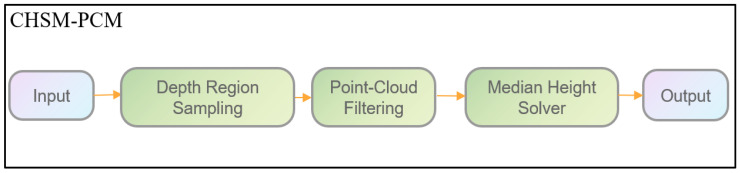
Architecture of the proposed CHSM-PCM.

After achieving continuous and stable tracking of corn ears, the Corn Height Solving Module based on Point-Cloud Median (CHSM-PCM) was applied to each tracked target to estimate its height, as illustrated in Figure 18. The predicted bounding boxes obtained from tracking were used as input, and the corresponding depth pixels within each box were back-projected to form a local point cloud. Depth outliers were removed, and the spatial height of each corn ear relative to the camera was computed as the median along the height axis of the point cloud. By incorporating the camera’s extrinsic parameters, including installation height and tilt angle, the absolute height of each corn ear relative to the ground was obtained, enabling reliable height estimation for all target ears in each frame.

### 4.6. Experimental Settings

#### 4.6.1. Parameter Settings

In this study, the deep learning hardware platform for the fresh corn ear detection task consisted of a laptop equipped with an Intel Core i7-11700 CPU (8 cores, Intel Corporation, Santa Clara, CA, USA), 16 GB of RAM, and an NVIDIA GeForce GTX 4060 GPU (16 GB VRAM, 3584 CUDA cores, NVIDIA Corporation, Santa Clara, CA, USA). A single GPU was used during training, with an average iteration time of approximately 30 s per epoch. The software environment included Python 3.8.8, PyTorch 1.9.0, torchvision 0.10.0, CUDA 11.1, and OpenCV-python 4.5.3.

The network input size was set to 640 × 640 pixels, with a batch size of 4 and a total of 300 training iterations. Stochastic gradient descent (SGD) served as the optimizer, with a momentum of 0.937, weight decay of 5 × 10^−4^, and an initial learning rate of 0.01. A cosine annealing schedule was applied to dynamically adjust the learning rate and improve training stability. During training, data augmentation was performed, including random horizontal flipping, rotation (±15°), brightness/contrast adjustment, random cropping, and HSV color space perturbation, to enhance model generalization. All models used for corn ear detection were trained on the same dataset with identical parameters to ensure fairness and consistency in comparative evaluations.

#### 4.6.2. Evaluation Metrics

In the field of fresh maize ear detection, commonly used performance evaluation metrics include IoU (Intersection over Union), Precision, Recall, and mAP (mean Average Precision). In object detection tasks, a target is generally considered successfully detected when IoU exceeds 0.5; therefore, an IoU threshold of 0.5 was adopted in this study. True Positives (*TP*) are defined as the number of positive samples correctly identified as maize ears, whereas False Positives (*FP*) denote the number of negative samples incorrectly classified as maize ears. True Negative (*TN*) denotes the number of negative samples correctly recognized as non-maize ears, and False Negative (*FN*) represents the number of actual maize ears that were not detected. *Precision* is calculated as the proportion of correctly identified maize ears among all predicted maize ears, while *Recall* is calculated as the proportion of correctly identified maize ears among all actual maize ears. The formulas for these metrics are expressed as follows:

(18) $$Precision=\frac{TP}{TP+FP}$$

(19) $$Recall=\frac{TP}{TP+FN}$$

In this study, model performance is comprehensively evaluated using multi-scale and multi-threshold mean Average Precision (mAP) metrics, including mAP@[0.5: 0.95], mAP@0.5, and mAP@0.75, along with average precision (AP) for targets of different sizes according to the COCO standard. During mAP calculation, the confidence threshold for predicted boxes is set to 0.25, and non-maximum suppression (NMS) with a threshold of 0.45 is applied to remove duplicate detections.

Furthermore, to comprehensively assess the accuracy of the corn ear height detection model integrating object detection with depth information, mean absolute error (MAE) and the Pearson correlation coefficient (r) are employed as evaluation metrics. MAE provides an intuitive measure of the average deviation between predicted and manually measured heights, expressed in the same units as corn ear height, thus serving as a suitable metric for evaluating height measurement errors. Manual measurements were performed using a steel tape measure (minimum scale 1 mm), from the ground at the center of the inter-row space to the midpoint of each ear. Each sample was independently measured twice by two experimenters, and the mean of the two measurements was used. The standard deviation of repeated measurements was less than 0.5 cm, confirming the reliability of the ground-truth heights. The Pearson correlation coefficient quantifies the linear relationship between automated measurements and manual references, reflecting the consistency and stability of the predicted trends. This metric complements MAE by providing insight into trend agreement, thereby enabling a more comprehensive evaluation of model performance.

The specific formulas are as follows:

(20) $$MAE=\frac{1}{N}\sum_{i=1}^{N}\left|{\hat{y}}_{i}-y_{i}\right|$$

where ${\hat{y}}_{i}$ denotes the machine-measured height of the i−th sample, $y_{i}$ represents the corresponding manually measured height, and *N* is the total number of samples.

(21) $$r=\frac{\sum_{i=1}^{N}\left({\hat{y}}_{i}-\hat{y}\right)\left(y_{i}-\bar{y}\right)}{\sqrt{\sum_{i=1}^{N}{\left({\hat{y}}_{i}-\bar{\hat{y}}\right)}^{2}}\sqrt{\sum_{i=1}^{N}{\left(y_{i}-\bar{y}\right)}^{2}}}$$

$\bar{\hat{y}}$ and $\bar{y}$ denote the mean values of the machine-measured and manually measured heights, respectively, and *r* ranges from −1 to 1, with values closer to 1 indicating a stronger linear correlation between the two sets of measurements.

## 5. Conclusions

This study integrates RGB and depth image data to address the challenges of corn ear detection in complex fresh corn field environments and proposes a corn ear height measurement model based on YOLO-HAMDF and DI-DeepSORT. Compared with conventional detection methods, the model employs the innovatively designed TBDA multi-scale dynamic convolution module, GLSA global-local feature aggregation module, and the improved AQE detection head, integrated with the DI-DeepSORT tracking module, thereby significantly enhancing both ear recognition accuracy and height measurement precision. The main conclusions are as follows:

(1) The synergistic integration of TBDA, GLSA, and AQE allows the model to achieve enhanced feature representation and discriminative capability in complex backgrounds and densely distributed corn ear scenarios. Under the combined effect of these modules, the model attains a detection accuracy of 97.01%, outperforming other state-of-the-art methods.
(2) By incorporating depth information from the Intel RealSense camera, non-target row ears can be effectively filtered, thereby reducing interference from non-target ears and enhancing both computational efficiency and practical applicability of the system.
(3) The integration of the IDA Kalman filter with the DBC-Net feature extraction network facilitates more stable cross-frame matching. When paired with the YOLO-HAMDF model for height measurement, the MAE is reduced to 3.21 ± 0.05 cm, and the Pearson correlation coefficient increases to 0.92 ± 0.01. This effectively mitigates detection box jitter caused by occlusion or detection fluctuations, providing continuous and reliable input for height curve extraction, further enhancing the overall stability and accuracy of the measurement.

## Figures and Tables

**Table 1:** Ablation studies on the FCE-GBD Dataset.

Baseline	TBDA ^1^	GLSA ^2^	AQE ^3^	DI- Deep SORT ^4^	mAP0.5 (%)	MAE (cm)	Pearson r	FLOPs (G)	Latency (ms)	FPS
√	×	×	×	×	93.21 ± 0.18	6.52 ± 0.12	0.83 ± 0.01	**86.9**	**32.1 ± 0.4**	**31 ± 0.4**
√	√	×	×	×	96.35 ± 0.12	5.26 ± 0.08	0.86 ± 0.01	87.4	33.5 ± 0.5	30 ± 0.5
√	×	√	×	×	96.19 ± 0.15	5.19 ± 0.10	0.85 ± 0.01	87.3	33.2 ± 0.5	30 ± 0.5
√	×	×	√	×	95.21 ± 0.10	4.15 ± 0.07	0.90 ± 0.01	87.1	32.8 ± 0.4	30 ± 0.4
√	×	×	×	√	93.21 ± 0.18	5.56 ± 0.09	0.84 ± 0.01	87.2	33.0 ± 0.4	30 ± 0.4
√	√	√	×	×	96.80 ± 0.10	4.90 ± 0.07	0.87 ± 0.01	87.8	34.6 ± 0.4	29 ± 0.4
√	×	√	√	×	96.75 ± 0.10	4.05 ± 0.06	0.91 ± 0.01	87.5	33.9 ± 0.4	29 ± 0.4
√	√	×	√	×	96.90 ± 0.09	4.00 ± 0.05	0.91 ± 0.01	87.6	34.2 ± 0.5	29 ± 0.4
√	√	√	√	×	**97.01 ± 0.08**	3.50 ± 0.05	0.92 ± 0.01	88.0	35.3 ± 0.5	28 ± 0.4
√	√	√	√	√	**97.01 ± 0.08**	**3.21 ± 0.05**	**0.94 ± 0.01**	88.3	36.5 ± 0.5	28 ± 0.4

^1^ Three-Branch Dynamic Attention module. ^2^ Global-Local Selective Aggregation module. ^3^ Adaptive Quality Estimator. ^4^ DBC-IDA DeepSORT. “√” = module present, “×” = module absent.

**Table 2:** Comparison of Detection Performances.

Method	mAP@[0.5:0.95] (%)	mAP (%)	mAP@0.75 (%)	AP_S (%)	AP_M (%)	AP_L (%)	Parameter (M)	FLOPs (G)
Faster R-CNN	74.32	79.24	68.50	52.1	76.3	88.5	41.12	216.30
Cascade R-CNN	75.20	79.50	69.20	53.8	76.9	88.5	68.93	244.10
YOLO5-L	85.12	89.47	80.33	68.5	84.7	92.1	46.11	107.6
YOLO8-L	88.50	92.35	84.20	72.0	87.5	94.0	43.61	164.82
RT-DETRv3	87.20	91.50	83.10	70.5	86.0	93.0	52.0	180.0
Swin-T	88.10	92.70	83.90	65.0	87.0	94.5	60.0	210.0
YOLO11-L	89.21	93.21	85.50	73.2	88.2	94.5	**25.3**	**86.9**
YOLO13-L	90.05	94.10	86.70	74.0	89.0	95.0	28.0	92.0
YOLO-HAMDF	**93.50**	**97.01**	**91.20**	**82.1**	**93.0**	**97.5**	26.4	89.1

**Table 3:** Comparison of Tracking Performances.

Tracker	MOTA (%)	IDF1 (%)	ID Switch	FPS
DI- DeepSORT	**92**	**90**	**15**	30
DeepSORT	88	85	30	**31**
ByteTrack	86	82	40	22
StrongSORT	89	87	20	15

**Table 4:** Comparison of Height Measurement Performances.

Detectors	Trackers	MAE (cm)	Pearson r	Latency (ms)	FPS
Swin-T	StrongSORT	3.98 ± 0.07	0.875 ± 0.02	53.0 ± 0.8	18.9 ± 0.3
Swin-T	DI-DeepSORT	3.91 ± 0.07	0.88 ± 0.02	49.9 ± 0.7	20.0 ± 0.3
YOLO13	StrongSORT	3.60 ± 0.06	0.895 ± 0.01	46.0 ± 0.7	21.7 ± 0.3
YOLO13	DI-DeepSORT	3.51 ± 0.06	0.90 ± 0.01	42.9 ± 0.6	23.3 ± 0.3
YOLO-HAMDF	StrongSORT	3.28 ± 0.05	0.915 ± 0.01	38.5 ± 0.6	26.0 ± 0.4
YOLO-HAMDF	DI-DeepSORT	**3.21 ± 0.05**	**0.92 ± 0.01**	**35.4 ± 0.5**	**28 ± 0.4**

## Data Availability

The datasets generated and analyzed during the current study, including RGB images, depth maps, and annotation files of fresh corn ears (FCE-GBD Dataset), are publicly available at [https://github.com/why110054/corn] (accessed on 31 October 2025). The dataset is released for academic research purposes under a CC BY 4.0 license. Researchers can freely download and use the data, provided that appropriate citation to the original study is given.

## Abbreviations

The following abbreviations are used in this manuscript:

RGB	Red, Green, Blue
CNN	Convolutional Neural Network
mAP	Mean Average Precision
IoU	Intersection over Union
MAE	Mean Absolute Error
TBDA	Three-Branch Dynamic Attention module
GLSA	Global-Local Selective Aggregation module
LQE	Location Quality Estimator
AQE	Adaptive Quality Estimator
DI-DeepSORT	DBC-IDA DeepSORT
DBC-Net	Dual-Branch Convolutional Network
IDA-Kalman	Independent-Dimension Adaptive Kalman Filter
CHSM-PCM	Corn Height Solving Module based on Point-Cloud Median
FPS	Frames per Second
CEHD	Corn Ear Height Detection framework
FCE-GBD Dataset	Fresh Corn Ear—Ground-Based RGB-D Dataset
